# The “Red Back”

**Published:** 1902-10

**Authors:** 


### THE "RED BACK."

---

### Some Specimen Letters.

---

PROCTOR, TEXAS, September 8, 1902.

*F. E. Daniel, M. D., Austin, Texas.*

DEAR SIR: Enclosed find one dollar for the "Red Back," the best journal in the South.

Fraternally yours,

J. J. EARGLE.

---

MARLIN, TEXAS, September 18, 1902.

*F. E. Daniel, Editor Texas Medical Journal.*

DEAR DOCTOR: Enclosed find check for "Red Back" for one year. The "Red Back" is the best journal in Texas or out of it for that matter. Yours fraternally,

J. G. MILLS.

---

GRAPELAND, TEXAS, September 17, 1902.

*Dr. F. E. Daniel, Editor Texas Medical Journal, Austin.*

DEAR SIR: Enclosed you will find one dollar. You ought to appreciate it. I made it by taking a long trip through the piney woods one dark night accompanied only by the "hoot of the owl" or the bark of the fox, to assist an old fellow's wife to perform one of the most important duties of life. And when I got through and was preparing for the long, lonesome return trip, he says, "Doc., what's the bill?" "Fifteen dollars," I says. "Here's one dollar," he says, "it's all yer gwine ter git." So I contribute it to Daniel's "Red Back."

Yours fraternally,

L. MERIWETHER.

---

DAWSON, TEXAS, September 22, 1902.

*F. E. Daniel, Editor Texas Medical Journal.*

DEAR DOCTOR: Enclosed find money order for "Red Back," which will place me away up. Keep up your fight against quacks and in favor of legislation regulating the practice of medicine till all the "cheap men" are driven out of Texas. Both of our candidates for representative in this county have promised me they would favor an amendment to present law which will drive out faith healers, Christian scientists and such ilks, who cannot stand a rigid examination before a competent board of examiners.

Yours fraternally,

B. W. D. HILL, M. D.

[The supreme court both of Alabama and Illinois have ruled that an "osteopath" *practices medicine,* within the meaning of the law, drugs or no drugs. We will try to get the Texas Legislature to amend our practice act to conform to that decision.—EDITOR.]

---

SABINE PASS, TEXAS, September 7, 1902.

*F. E. Daniel, M. D., Austin, Texas.*

DEAR DOCTOR: I believe when a man is doing a good work for the medical profession or for any other good cause he ought to know that his labors are appreciated by those for whom he labors. I have always, since I have been in the profession, been a great reader and lover of medical journals, and when I say that I consider the TEXAS MEDICAL JOURNAL one of the very best medical publications in the United States I do not mean to flatter you, but I mean only to express my sincere opinion. It is a journal the profession in Texas are proud of.

I notice in the last issue the death of Dr. Osborn, of Cleburne. He was, I think, one of our great men. A noble old veteran who deserves a monument more durable than brass to his memory. I always loved to read his papers, and never failed to derive benefit from them.

I enjoy very good health, but am a cripple, the result of an injury I received several months ago.

What is your opinion about mosquitoes being the source of malaria? I am not a convert to the theory. If the theory is true, the people on the Texas coast would have nothing in them but malaria.

Yours truly,

A. N. Perkins.

[The venerable Dr. Perkins was for many years quarantine officer at Sabine Pass. He has read the Journal from Volume I, No. 1 (1885), to date.—Editor.]

